# Diagnostic performance of CareStart™ malaria HRP2/pLDH (Pf/pan) combo test versus standard microscopy on *falciparum* and *vivax* malaria between China-Myanmar endemic borders

**DOI:** 10.1186/1475-2875-12-6

**Published:** 2013-01-07

**Authors:** Sun Xiaodong, Ernest Tambo, Wei Chun, Cheng Zhibin, Deng Yan, Wang Jian, Wang Jiazhi, Zhou Xiaonong

**Affiliations:** 1 Yunnan Institute of Parasitic Diseases, Yunnan Center for Malaria Research, Institute of Vector and Pathogen Biology of Dali University, Puer, Yunnan, 665000, China; 2 National Institute of Parasitic Diseases, Chinese Center for Diseases Control and Prevention, WHO Collaborating Center on Malaria, Schisostomiasis and Filariasis, Shanghai, 200025, China; 3 Institute of Basic Medical Sciences, Chinese Academy of Medical Sciences and Peking Union Medical College, Beijing, 100005, China; 4 Tengchong Center for Disease Control and Prevention, Tengchong, Yunnan, 679100, China

**Keywords:** CareStart™, Diagnostic, Malaria, Microscopy, Performance, *Plasmodium*, Standard

## Abstract

**Background:**

Rapid diagnostic test (RDT) is becoming an alternative way of establishing quickly the diagnosis of malaria infections, by detecting specific malaria antigens in suspected patients’ blood between the China-Myanmar endemic borders areas, towards achieving the National Malaria Elimination programme by 2020. The objective of this study is to evaluate the performance of CareStart™ Malaria Pf/Pan RDT kit for the diagnosis of malaria infections in suspected patients. Blood examination by microscopy was taken as gold standard to evaluate CareStart™ kit’s sensitivity, specificity and predictive value and corrected with PCR assay.

**Results:**

Overall 126 of 241 (52.28%) malaria cases were detected by microscopy compared to 115 of 241(47.72%) CareStart™ kit and 128 of 241 (53.11%) PCR corrected assay. CareStart™ kit’s sensitivity and specificity for the diagnosis of malaria were 89.68% and 98.26% respectively, compared to standard microscopy, whereas the sensitivity and specificity for *falciparum* malaria were 88.52% and 98.26%, and for *vivax* malaria: 90.77% and 100%. The CareStart™ positive predictive values were 98.26% (93.88-99.52%, 95% CI) compared to 100% (96.77-100%, 95% CI) for PCR-corrected, and the negative predictive values of 89.68% (83.15-93.87%, 95% CI) were the same in microscopy as PCR-corrected. The diagnostic accuracy of CareStart™ kit versus microscopy and PCR were 93.78% (89.99-96.19%, 95% CI) and 94.61% (90.99-96.82%, 95% CI) respectively. The likelihood of diagnostic of malaria positive was almost similar between microscopy and CareStart™ kit, with an entropy reduction of 60.0% compared to a weak likelihood of misdiagnosis of 0.10 (0.09-0.12, 95% CI), with an entropy reduction of 36.01%.

**Conclusion:**

The accuracy of CareStart™ kit is comparable to gold standard microscopy in these areas, it is easy to perform and suitable for cross-border diagnosis and monitoring of local or imported malaria patterns by any local health staff in endemic remotes.

## Background

Malaria remains unevenly endemic across Yunnan The CareStart™ *P*.*f*/*P*.*v* Combo province, Southeast China. Within Yunnan, malaria occurs mainly at the border areas, in the drainage areas of the Yuanjiang River and amongst the high population of motile ethnic minority groups at risk living in forests and on forest fringes for economic reasons, on the China-Myanmar borders [1]. While parasite-based diagnosis is increasing, most suspected cases of malaria are not properly identified, with accurate diagnosis and disease monitoring consequently remaining elusive [2].

Although malaria cases and deaths have dropped substantially over the past decade, efforts must be intensified in the malaria-endemic communities to abolish the public threat of malaria by prompt diagnosis and effective treatment [3]. However, limitations of comparative field trials and the heterogeneous nature of malaria transmission has limited the availability of good quality performance diagnostic that the National Malaria Elimination programme requires for a surveillance response system and to make informed decisions and implementation towards malaria elimination by 2020.

Technical support for malaria prevention and control in China is provided at national level by the National Institute of Parasitic Diseases, Chinese Center for Disease Control and Prevention (CDC), based in Shanghai. The strategies of the National Malaria Elimination programme 2010–2020 include greater access to early diagnosis and prompt treatment coverage in remote areas, free distribution of long-lasting, insecticide-treated bed nets (LLINs), strengthening quality and regulatory mechanisms for anti-malarial drugs, strengthening malaria surveillance and information systems, epidemic preparedness, increasing the awareness of malaria, involvement and empowerment, basic and applied research on *Plasmodium vivax* and *Plasmodium falciparum*, whilst monitoring the indicators for malaria elimination [3]. Moreover, World Health Organization (WHO) recommends that malaria case management be based on parasite-based diagnosis in all cases, with the exception of young children in areas of high transmission and where lack of resources or need for urgent response temporarily limits its applications [1-4] The use of antigen-detecting rapid diagnostic tests (RDTs) forms a vital part of this strategy, providing the possibility of parasite-based diagnosis in areas where good quality microscopy cannot be maintained [4]. There are numerous malaria RDTs that are commercially available in many formats, including plastic cassettes, cards or dipsticks, and quality depends on manufacturer as well as storage conditions [5]. The variation depends on malaria antigen targeted for detection in blood flowing along a membrane containing specific anti-malaria antibodies. Previous studies from clinical trials have found that HRP-2 based *P*. *falciparum*-specific tests generally have greater sensitivity (over 90%) than the pLDH-based tests when compared with microscopy in clinical cases, whilst sensitivity of pLDH tests for non-*P*. *falciparum* species was low [6,7]. Specificity of both types of tests was reported to be good (>85%). Despite the encouraging results from the RDT trials in clinical cases, there is limited information about the sensitivity, specificity and predictive value in the endemic China-Myanmar malaria population where laboratory facilities are not available. Reports of wide variation in accuracy of malaria RDTs in published field trials usually compared with light microscopy as a gold standard [8-10]. The CareStart™ *P*.*f*/*P*.*v* combo test (detecting histidine-rich protein (pHRP-2) and *P*. *vivax*-specific lactate dehydrogenase (pLDH) has been evaluated in a few field settings, including higher sensitivities for *P*. *falciparum* (99.4%) reported in Ethiopia with *P*. *falciparum* samples with parasite densities above 100/μl, [11], sensitivities for *P*. *falciparum* low values at parasite densities <100/μl (60.0%) and increasing sensitivity at higher parasite densities (100% at >500/μl) reported in Madagascar [12], and sensitivities for the detection of *P*. *vivax* were significantly higher than those found in Ethiopia with pLDH (pan) (91.0%), but similar results for the CareStart™ malaria pLDH (pan/Pf) reported in Myanmar [13]. Similarly, some studies evaluating other RDTs in non-endemic countries reported similar sensitivities as those found for the CareStart™ malaria HRP-2/pLDH (Pf/pan) combo test but a different degree of sensitivity and specificity for *P*. *falciparum* ranging from 87.5-99.0% [6-14] and for *P*. *vivax*, RDTs detecting pan-pLDH showed sensitivities ranging from 62.0-95.0%, [10-15] compared to 46.0%-93.0% for those RDTs targeting aldolase [16]. The increase of sensitivities at higher parasite densities is a well known phenomenon with breakpoints around 100/μl (*P*. *falciparum*) and 500/μl (*P*. *vivax*) [6,13,17,18].

The aim of the study was to evaluate the performance of CareStart™ malaria Pf/pan test kit in early diagnosis for prompt effective case management of malaria.

## Methods

### Study design

The CareStart™ malaria HRP-2/pLDH (Pf/pan) combo test was evaluated in suspected patients attending TengChong CDC, China and Health Unlimited clinic in Laiza City, Kachin State, Myanmar, near the China-Myanmar border. Standard microscopy method was done for all thick smears corrected by polymerase chain reaction (PCR) assay. The assessment of the diagnostic performance was performed in comparison the CareStart™ (CS) kit with gold standard microscopic results, corrected by PCR assay.

### Blood sample collection

The study was conducted in July to December, 2011 in TengChong CDC, China and Health Unlimited clinic in Laiza City, Kachin State, Myanmar, near the China-Myanmar border on all suspected patients. Individual biodata and malaria history in the previous one year were documented from each suspected case as well as an informed consent. All patients had venous blood samples collection for microscopy and CS kit evaluation and also samples were collected in EDTA and stored at −20°C condition for PCR analysis.

### Ethical clearance

The study was reviewed and approved by the Ethical Committee of Yunnan Institute of Parasitic Diseases, Yunnan province and Health Ministry of Kachin State of Myanmar. All patients provided informed consent before admission into the study.

### Standard microscopy evaluation

Duplicate thin and thick slides were made from all blood, stained using 5% Giemsa stain and dyed for 30 minutes, read under x100 electron microscopy. The malaria microscopic examination was performed by two independent experienced microscopists, for identification of the malaria parasite species. The number of parasites was counted against 200 leucocytes and quantification of parasite density was estimated by assuming 8,000 leucocytes/μl blood. Samples were considered negative when no parasite was detected after examining 100 microscopic fields [19]. In order to check for interobserver difference, a double-blind cross reading of a random 50 blood slides was performed by a senior microscopist.

### CareStart™ malaria kit assessment

The CS RDT kit contains a membrane strip, which is pre-coated with two monoclonal antibodies as two separate lines across a test strip. One monoclonal antibody (test line 2) is pan-specific to lactate dehydrogenase (pLDH) of the *Plasmodium* species (*P*. *falciparum*, *P*. *vivax*, *Plasmodium malariae* and *Plasmodium ovale*) and the other line (test line 1) consists of a monoclonal antibody specific to histidine-rich protein 2 (HRP2) of the *P*. *falciparum* species. The conjugate pad is dispensed with monoclonal antibodies, which are pan-specific to pLDH and *P*. *falciparum* specific to HRP2. The CS RDT kit is designed for the differential diagnosis between *P*. *falciparum* and other pan-specific species. The CS RDT kit was supplied by the China-England Advanced Communication Project and is produced by American Access Bio Company; batch number C201R, period of validity to February 2012. The test reproducibility was evaluated by testing 15 samples representing all species at variable parasite densities on three consecutive occasions in Teng Chong CDC.

### CareStart™ malaria test procedures

Tests were performed according to manufacturer’s instructions. Readings were carried out at daylight, assisted by a standard electric bulb, by three health worker-observers performing and interpreting the test results. The first observer performed readings at 20 minutes, reading time recommended by the manufacturer, followed by observers 2 and 3 within the next 10 minutes. The observers were blinded to each other’s readings and to the results of microscopy and PCR assay.

In the case when no control line appeared, the test was considered invalid and was repeated. To score line intensities, a scoring system of five categories was used as defined previously [7]: none (no line visible), faint (barely visible line), weak (paler than the control line), medium (equal to the control line) or strong (stronger than the control line).

The test results were based on consensus agreement, which meant that an identical result read by at least two out of three health worker-observers was withheld. In the case of no consensus, the results of the first reader were considered. To assess inter-observer agreement, results of positive and negative readings as well as line intensities were considered.

### Test detection and validation

The sensitivity and specification of CS RDT kit were performed by random selection of *P*. *falciparum* and *P*. *vivax* cases, and negative blood samples for 10 times repeat tests, and the results estimated following the manufacturer specification.

### *Plasmodium* species identification

All cases were identified by microscopy and analysed by real-time PCR for detection of the 18 s rRNA of malaria parasite for confirmation, species identification and determination of parasite density, as described previously [20]. In the case of discordant results between microscopy and PCR, the results of PCR were used as the standard method.

### Data analysis

All data were processed using Excel to build database and Wilson score software was used for analysis. Sensitivity, specificity and predictive values were calculated separately for *P*. *falciparum* and the non-*falciparum* species with 95% CI.

The Pearson Chi-square test was used to determine significance of results, or in the case of a small sample size, a two-tailed Fisher’s exact test. A p-value <0.05 was considered statistically significant.

## Results

### General characteristics of patients

A total of 241 blood samples from suspected patients were analysed for malaria parasites by the CS RDT kit and the results were compared with results from gold standard microscopy, corrected by PCR assay. The male: female ratio was 3.54:1. The mean age was 29.62 ± 11.21 years (range 3–58 years old) of which the majority 89.21% (215) were older than 18 years and only four patients (1.66%) were under five years. The microscopic results indicated that 52.28% (126 of 241) of patients were infected with malaria. Among the malaria-positive patients, 51.58% (65 of 126) harboured *P*. *vivax*, 43.63% (55 of 126) had *P*. *falciparum*, and 4.76% (six of 126) mixed infections of *P*. *vivax* and *P*. *falciparum*. There were only two cases of missed diagnosis observed by microscopy.

The geometric mean parasite density was 5,990, ranged (48~377143/μl. The parasite density distribution showed that 3.18% cases were <100 parasites/μl, 11.90% cases were between 100~1000/μl and 84.95% cases were >1,000 parasites/μl.

The median parasite density in 13 missed cases by CS was 348/μl, ranged from 48 ~ 22,688/μl, of which four cases were <100 μl, three cases between 100~1,000/μl and six cases were >1,000 parasites/μl (see Table 1).

**Table 1:** General characteristics of patients

**Total**		**241**
Mean age (range) (years)		29.62 ± 11.21 (3–58)
Age group (%)	≤5 years old	4 (1.66%)
	5–18 years old	22 (9.13%)
	>18 years old	215 (89.21%)
Gender (%)	Male	188 (78.01%)
	Female	53 (21.99%)
Geometric mean parasite density (range)/μl		5,990 (48~377143)/μl
Parasite density distribution	<100/μl	4 (3.18)%
	100 ~ 1,000/μl	15 (11.90%)
	>1,000/μl	107 (84.92%)

### Comparison of CareStart™ kit, standard microscopy and polymerase chain reaction results

Two (1.58%) positive cases initially misdiagnosed by microscopy were identified by PCR assays and CS RDT kit. There was no significant difference between the sensitivity of PCR and standard microscopy, however the specificity of PCR assay was 100% against 98.26% for microscopy. The diagnostic accuracies of both diagnostic methods were comparable. The likelihood of ratio of a positive test and negative test were 51.57 (19.32-137.7) and 0.11 (0.09, 0.12) respectively. The negative entropy after a positive microscopic test was 60.44%, whereas the microscopy and PCR entropy reduction after a negative test were 36.1% and 35.96% respectively. There was no significant difference between the Cohen’s kappa values of microscopy compared to PCR assay (see Table 2).

**Table 2:** Comparison with microscopy and polymerase chain reaction results

**CS RDT kit**	**Microscopy**		**PCR**	
	**Positive**	**Negative**	**Positive**	**Negative**
Positive	113	2	115	0
Negative	13	113	13	113
Sensitivity (95% IC)%	89.68 (83.15, 93.87 )		89.84 (83.4, 93.97 )	
Specificity (95% IC)%	98.26 (93.88, 99.52 )		100 (96.71, 100 )	
Positive Predictive Value (95% IC)%	98.26 (93.88, 99.52 )		100 (96.77, 100 )	
Negative Predictive Value (95% IC)%	89.68 (83.15, 93.87 )		89.68 (83.15, 93.87 )	
Diagnostic Accuracy	93.78 (89.99, 96.19 )		94.61 (90.99, 96.82 )	
Likelihood ratio of a Positive Test	51.57 (19.32 – 137.7)		-	
Likelihood ratio of a Negative Test	0.11 (0.09, 0.12)		0.1 (0.09 0.12)	
Diagnostic Odds	491.1 (108.3 – 2226)		-	
Cohen’s kappa (Unweighted)	0.88 (0.75 – 1.00)		0.89 (0.77, 1.02)	
Entropy reduction after a Positive Test%	60.44		-	
Entropy reduction after a Negative Test%	36.01		35.92	
Bias Index	−0.04		−0.05	

### CareStart™ malaria test results

The CS kit results indicated that 47.72% (115/241) of the patients were infected with malaria. Among the positive samples, malaria species identified showed that *P*. *vivax* represented 51.30% (59/115), *P*. *falciparum* or *P*. *falciparum* mixed other species was present 48.70% (56/115). Two cases microscopically negative were CS kit *P*. *falciparum* positive whereas 13 cases (six cases of *P*. *vivax* and seven cases of *P*. *falciparum*) originally detected by microscopy were not detected by CS RDT kit. The summary results of microscopy and CS kit results are showed in Table 3.

**Table 3:** CareStart™ and PCR sensitivity, specificity and predictive values

**CS Diagnostic Kit**	**Microscopy**				**PCR**			
	P. falciparum*		***P. vivax***		P. falciparum*		**P.*vivax***	
	**Positive**	**Negative**	**Positive**	**Negative**	**Positive**	**Negative**	**Positive**	**Negative**
Positive	54	2	59	0	56	0	59	0
Negative	7	113	6	115	7	113	6	113
Sensitivity (95% IC)%	88.52 (78.16, 94.33)		90.77 (81.29, 95.7)		88.89 (78.8, 94.51)		90.77 (81.29, 95.7)	
Specificity (95% IC)%	98.26 (93.88, 99.52)		100 (96.77, 100)		100 (96.71, 100)		100 (96.71, 100)	
Positive Predictive Value (95% IC)%	96.43 (87.88, 99.02)		100 (93.89, 100)		100 (93.58, 100)		100 (93.89, 100)	
Negative Predictive Value (95% IC)%	94.17 (88.45, 97.15)		95.04 (89.6, 97.71)		94.17 (88.45, 97.15)		94.96 (89.44, 97.67)	
Diagnostic Accuracy (95% IC)%	94.89 (90.57, 97.29)		96.67 (92.92, 98.46)		96.02 (92.02, 98.06)		96.63 (92.84, 98.45)	
Cohen’s kappa (Unweighted)	0.89 (0.74, 1.03)		0.93 (0.78,1.07)		0.91 (0.76, 1.06)		0.93 (0.78, 1.07)	

*Plasmodium falciparum.

### Sensitivity, specificity and predictive values of CareStart™ malaria kit

The CareStart™ *P*.*f*/*P*.*v* Combo test sensitivity ranged between 88.55 - 90.77% and specificity ranged 98.26 - 100% (95%CI) when compared to gold standard microscopic blood films for detection of malaria. The mean CS malaria *P*.*v*/*P*.*f* rapid kit sensitivity was 89.68% (83.15-93.97%, 95% CI) and mean specificity was 98.26% (93.88-100%, 95% CI). The positive predictive value was 98.26% (93.88-99.52, 95% CI) and negative predictive value of 89.68% (89.68-3.87%, 95% CI) when compared to traditional blood films for *falciparum* and *vivax* malaria detection. The diagnostic accuracy of CS compared to microscopy was 93.78% (89.99-96.19%) (see Table 3).

### Results of polymerase chain reaction assay

Real-time PCR for diagnosis of malaria, and identification of parasite species showed a difference in *P*. *falciparum* and *P*. *vivax* sensitivity of 88.89% and 90.77%, but similar specificity species identification of 100%. The assay confirmed 128 *Plasmodium* cases of which 49.22% (63/128) were *falciparum* against the predominant 50.72% (65/128) *vivax* malaria in the areas. In contrast, CS diagnosis had a lower specificity to *P*. *vivax* of 98.26%, but same specificity for *P*. *falciparum* when compared with corrected PCR assay results, as shown in Table 3.

## Discussion

The important finding of this study was that the CS kit had sensitivity of 89.68% and specificity of 98.26% compared to the gold standard microscopy method for detection of malaria. The CS sensitivity for the detection of *P*. *falciparum* was 98.3% and 99.3% in samples with parasite densities above 100/μl and 1,000/μl respectively whereas the sensitivity for *P*. *vivax* was 97.6% for parasite densities above 500/μl. No positive result occurred among the *Plasmodium*-negative samples, which is consistent with the recent study of CS kit in Ethiopia [21]. RDTs detecting *P*. *vivax*-specific pLDH CS kit based on specific monoclonal antibody, detected histidine-rich protein 2(HRP-2, test line 1) and four kinds of lactic acid dehydrogenase of *Plasmodium* in human blood (pLDH, test line 2) in a card detection device, aseptic and single packing. The comparison of the sensitivity and specificity of the CS-RDTs *versus* microscopy corrected by PCR assay (Table 3) indicated that the detection rate was relatively similar for *P*. *vivax* and *P*. *falciparum*, indicating that the microscopic training performed had a high efficiency on the performance microscopists. This kit showed no need of sophisticated equipment and facilities, and was easy to operate. Its applicability and rapidity in diagnosis could be of additional value for both *falciparum* and *vivax* malaria detection and prompt case management as well as cross borders malaria monitoring in the China-Myanmar endemic areas in the National Malaria Elimination programme.

In total, 241 kits, and 20 kits for quality assurance and control, were used in this study without the invalid case, specificity and sensitivity of this batch kit was 100%, which implied the quality was stable. However, the false negative rate of (10.4%) 13/126 documented in the study can be explained only partly by the CS threshold level of >100 parasites/μl and probably the CS storage conditions and duration in the field resulted in loss of sensitivity, since the quality assurance and control testing was 100%.

As shown in Tables 2 and 3, the diagnostic standards of kit were highly specific and sensitive, indicating that kit was valuable for malaria diagnosis.

The CareStart™ *P*.*f*/*P*.*v* Combo (detecting HRP-2 and *P*. *vivax*-specific pLDH) test has been evaluated in Ethiopia [11-25] compared to this study which reported higher sensitivity for *P*. *falciparum* (99.4%). No clear reason could be given for increasing sensitivity and possibly included exclusively *P*. *falciparum* samples with parasite densities above 100/μl, which is above the detection threshold of most RDTs [22]. Also, CareStart™ malaria pLDH (Pf/pan) combo test has been evaluated in a field study in Madagascar which reported sensitivities for *P*. *falciparum* low values at parasite densities <100/μl (60.0%) and increasing sensitivity at higher parasite densities (100% at >500/μl) [23].

The two products (CareStart™ malaria pLDH (Pf/pan) combo test) and CareStart™ brand (malaria pLDH (pan)), evaluated in Myanmar, reported sensitivities for the detection of *P*. *vivax* were significantly higher than those found in Ethiopia with pLDH (pan) (91.0%), but results were similar for the CareStart™ malaria pLDH (pan/Pf) [13-25].

A study in The Philippines compared the results of the ICT malaria P.f/P.v RDT with microscopy and found a high discordant rate involving cases positive for *P*. *falciparum* by the RDT, but negative by microscopy [24].

The CareStart™ malaria pLDH (Pf/pan) combo test field study in Madagascar reported sensitivities for *P*. *falciparum* that are comparable to the present study, but there were only nine *P*. *vivax* samples included, making comparison difficult [12]. Moreover, the CareStart™ brand (malaria pLDH (pan)) has also been evaluated in Myanmar with similar results obtained across the China-Myanmar malaria endemic borders [13].

When PCR was the reference method, the microscopic method showed a low specificity (88.2%). The *P*. *vivax* and *P*. *falciparum* positive and negative predictive values of CS were 100% and 94.96% compared to 100% and 94.17% respectively. In addition, *P*. *vivax* diagnostic accuracy of CS was 96.67% compared to 94.89% for *P*. *falciparum*. The differential explanation could be the lack of differentiation of other species of *Plasmodium* species as reported in Thailand [6]. However, the two blood slides from the samples could not show malaria parasites microscopically. The negative predictive values of CS observed could not be directly explained but may be attributed to possible genetic heterogeneity of HRP2 as well as possible geographic variations in malaria antigens.

This study recorded two misdiagnosed microscopic cases, corrected using PCR assay, whereas 13 cases were not detected by CS kit. The reported sensitivities for the detection of *P*. *vivax* were significantly higher than those found in the present study in case of the CareStart™ malaria pLDH (Pf/pan) (88.52%), but for the CareStart™ malaria pLDH (Pv/pan) they were in line with the previous findings (90.77%) [10,11].

Studies evaluating other RDTs in non-endemic countries report similar sensitivities as those found for the CareStart™ malaria HRP-2/pLDH (Pf/pan) combo test in this study for *P*. *falciparum* where they ranged from 87.5-99.0%, with one exception of 76.2%, whereas for *P*. *vivax*, RDTs detecting pan-pLDH showed sensitivities of 33.5% and 62.0%-95.0% [6-9]. The detection rates as demonstrated by WHO/FIND are slightly higher as compared to the sensitivities found in this study, but the differences were not statistically significant [4].

The specificity and sensitivity of CS kit were 89.68% and 98.26%, a difference not statistically significant than previous CS results in southwest Ethiopia (see Table 2) [11]. The sensitivity and specificity documented in this study can be accounted for by the long historical trends of anti-malarial usage, counterfeit, substandard and counter prescription contributing to the progression of selective pressure of malaria parasites in the blood stream and hence low parasitaemia below the CS kit detection threshold. The findings showed that CS usefulness in detection and case management of malaria in remote areas and on motile populations across borders has implications in the search for more efficient and sensitive diagnostic tools for parasite detection and case management in areas of low endemicity, as well as targeting gametocyte detection towards blocking the transmission, and monitoring malaria elimination.

## Conclusion

The study demonstrated that the CareStart™ malaria HRP-2/pLDH (Pf/pan) combo test performs satisfactory well for the detection of *P*. *falciparum* and *P*. *vivax* malaria infections in cross-border malaria. Hence, providing a rationale surveillance response system for malaria cross-border control and elimination programmes in conjunction with national malaria programmes is urgently needed for China’s National Malaria Elimination programme for 2020.

## Abbreviations

CS: CareStart™; HRP-2: Histidine-rich protein 2; pan-pLDH: Pan *Plasmodium*-specific parasite lactate dehydrogenase; PCR: Polymerase chain reaction; CI: Confidence interval; FIND: Foundation for innovative new diagnostics; Pf-pLDH: *Plasmodium falciparum*-specific parasite lactate dehydrogenase; pLDH: *Plasmodium*-specific parasite lactate dehydrogenase; Pv-pLDH: *Plasmodium vivax*-specific parasite lactate dehydrogenase; RDTs: Rapid diagnostic test; WHO: World health organization.

## Competing interests

The authors declare that they have no competing interests.

## Authors’ contributions

ET, SX and XZ participated in the design of the study, execution, statistical analysis and drafted the manuscript. WC, CZ, ET and DY performed the PCR assays. WJ, WJ collected the blood sample. All authors read and approved the manuscript.
